# Single-shot circular dichroism spectroscopy

**DOI:** 10.1038/s41377-022-00830-8

**Published:** 2022-05-10

**Authors:** Alina Karabchevsky

**Affiliations:** Ben-Gurion University of the Negev, School of Electrical and Computer Engineering, Department of Photonics and Electrooptics, Beer-Sheva, 8410501 Israel

**Keywords:** Circular dichroism, Sub-wavelength optics

## Abstract

Circular dichroism spectroscopy can be optimized to measure the scattering circular dichroism response of a single chiral nanostructure with a single shot.

Chiral objects—objects with the geometrical property of lacking any planes of symmetry, are widespread in nature, with some examples being DNA and sugars. The concept of chirality had already been discovered by Louis Pasteur in 1848, while investigating the enantiomers of a tartaric acid salt^1,2^, however, it was Lord Kelvin who coined the term chirality in 1893^2^ “I call any geometrical figure, or group of points, ‘chiral’, and say that it has chirality if its image in a plane mirror, ideally realized, cannot be brought to coincide with itself”.

Chirality was derived from the Greek word (χ∈ιρ or kheir) ‘ceir’ meaning hand because our hands, enantiomers, display a good example of chirality since they are non-superimposable mirror images of each other. One of the intriguing properties of chiral molecules is optical activity because chiral molecules can rotate the plane of polarization differently by interacting with the electric field differently. Chiral molecules are very similar to each other since they have the same components to them. The only thing that differs is their arrangement in space. As a result of this similarity, it is very hard to distinguish chiral molecules from each other when we try to compare their properties such as boiling points, melting points and densities. Since optical activity, is the unique property of chiral molecules, one can differentiate them via optical activity. When plane-polarized light is passed through one of the two enantiomers of a chiral molecule, that molecule rotates light in a certain direction. When the same plane-polarized light is passed through the other enantiomer, that enantiomer rotates light by the same amount but in the opposite direction.

It was the Dutch chemist J. H. van ‘t Hoff and the French chemist J. A. Le Bel who, independently of each other in 1874, discovered the tetrahedral arrangement of groups around the central carbon atom (van ‘t Hoff received the first Nobel Prize in Chemistry 1901, but for other discoveries). Thus the enzymes in our cells are chiral, as are other receptors that play an important part in cell machinery. This means that they prefer to bind to one of the enantiomers because the receptors are extremely selective; only one of the enantiomers fits the receptor’s site like a key that fits a lock - this metaphor comes from another Nobel Laureate in Chemistry, Emil Fischer, who was awarded the Prize in 1902. Later, in 2001, the Nobel Prize in Chemistry 2001 was divided, one half jointly to William S. Knowles and Ryoji Noyori “for their work on chirally catalyzed hydrogenation reactions” and the other half to K. Barry Sharpless “for his work on chirally catalyzed oxidation reactions”.

Despite these impressive results, however, chirality is not limited to molecular systems and may appear on all scales, ranging from spiral galaxies to nanostructures. Accompanying the development of nanoscience and technologies the fabrication and measurements of artificial chiral structures have been the subject of intense research, owing to the many potential applications in biology, pharmacy, chiral catalysis, light manipulation and light-matter interaction^3,4^. Many of these emerging applications require polarization dispersive imaging spectrometers for scattering circular dichroism spectroscopy of single chiral nanostructures. Even if circular dichroism spectroscopy is one of the most important tools in nanoscopic chiroptics for studying the 3D conformation of molecules and nanostructure, such as chirality of molecules, the conformation of proteins and chiroptical properties of nanostructures, there is a lack of a simple, fast and reliable method for measuring the circular dichroism responses of single nanostructures. To tackle this issue, the group of Weihua Zhang^5^, reported a polarization-dispersive imaging spectrometer that is capable of measuring the scattering circular dichroism response of a single chiral nanostructure with a single shot. Using this technique, the authors studied the scattering circular dichroism spectra of a model system, the vertically coupled plasmonic nanorod pair as shown in Fig. 1. From the experimental and theoretical study, it was concluded that the polarization-dispersive spectrometer measures the imaginary part of the nonlocal susceptibility of the nanostructure.

**Fig. 1:**
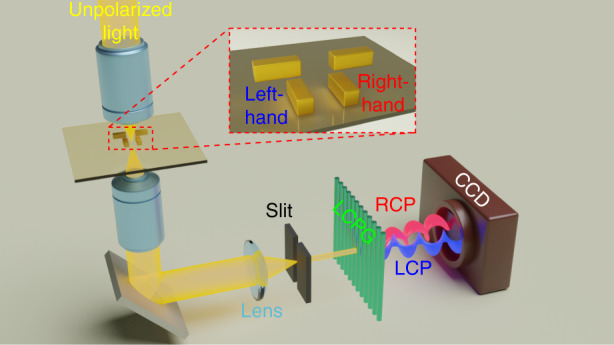
Schematics of the polarization-dispersive spectrometer for Scattering Circular Dichroism Spectroscopy. CCD coupled charged detector, LCPG liquid crystal polarization grating

The technical methodology demonstrated in this work uses polarization-dispersive imaging spectrometry applied to 3-dimensional Au nanorod structures assembled on DNA origami templates together with correlated scanning electron microscopic measurements, and is very likely to be used in other applications in the future. Another promising area of research will be to expand the genetic algorithm approach to use the wider range of tools in the general field of Machine Learning, Neural networks, for example, to predict the polarization of scattered fields and relate it to the polarization of the incident field and the chiroptical phenomena at the single nanostructure level. This appears a natural next step in the field, and with these techniques, it may even be possible to detect single DNA-assembled plasmonic chiral nanostructures and further correlate the spectra with their geometry using additional data from scanning electron microscope (SEM) images of the same structures.
